# Graphene oxide as an interface phase between polyetheretherketone and hydroxyapatite for tissue engineering scaffolds

**DOI:** 10.1038/srep46604

**Published:** 2017-04-20

**Authors:** Shuping Peng, Pei Feng, Ping Wu, Wei Huang, Youwen Yang, Wang Guo, Chengde Gao, Cijun Shuai

**Affiliations:** 1 The Key Laboratory of Carcinogenesis of the Chinese Ministry of Health, Xiangya Hospital, Central South University, 410008, China; 2 The Key Laboratory of Carcinogenesis and Cancer Invasion of the Chinese Ministry of Education, Cancer Research Institute, Central South University, 410078, China; 3 Hunan Key Laboratory of Nonresolving Inflammation and Cancer, Disease Genome Research Center, The Third Xiangya Hospital, Central South University, 410078, China; 4 State Key Laboratory of High Performance Complex Manufacturing, Central South University, 410083, China; 5 State Key Laboratory for Powder Metallurgy, Central South University, 410083, China; 6 College of Chemistry, Xiangtan University, 411105, China

## Abstract

The poor bonding strength between biopolymer and bioceramic has remained an unsolved issue. In this study, graphene oxide (GO) was introduced as an interface phase to improve the interfacial bonding between polyetheretherketone (PEEK) and hydroxyapatite (HAP) for tissue engineering scaffolds. On the one hand, the conjugated structure of GO could form strong π-π stacking interaction with the benzene rings in PEEK. On the other hand, GO with a negatively charge resulting from oxygen functional groups could adsorb the positively charged calcium atoms (C sites) of HAP. Consequently, the dispersibility and compatibility of HAP in the PEEK matrix increased with increasing GO content up to 1 wt%. At this time, the compressive strength and modulus of scaffolds increased by 79.45% and 42.07%, respectively. Furthermore, the PEEK-HAP with GO (PEEK-HAP/GO) scaffolds possessed the ability to induce formation of bone-like apatite. And they could support cellular adhesion, proliferation as well as osteogenic differentiation. More importantly, *in vivo* bone defect repair experiments showed that new bone formed throughout the scaffolds at 60 days after implantation. All these results suggested that the PEEK-HAP/GO scaffolds have a promising potential for bone tissue engineering application.

Biopolymer polyetheretherketone (PEEK) exhibits poor bioactivity and limited bone binding ability^1^,^2^ in spite of good biocompatibility, mechanical compatibility and processing properties^3^,^4^,^5^. Hydroxyapatite (HAP) is a bioceramic material that has good biocompatibility and bioactivity, and can form a strong bonding with bone tissue^6^,^7^,^8^. While the application of HAP alone for bone scaffold is restricted due to its brittleness and difficult processing^9^,^10^. The combination of biopolymer and bioceramic as scaffold material may exert their respective superiorities, and avoid their shortcomings^11^,^12^,^13^,^14^. However, there are very differences in their physical and chemical properties because biopolymer and bioceramic belong to the organic phase and inorganic phase, respectively^15^,^16^. Thus bioceramic is difficulty in homogeneously dispersing in the biopolymer matrix and the overall performance of their composite decreases. The introduction of the interface phase between biopolymer and bioceramic is a very effective method to enhance the compatibility between the two phases^17^,^18^,^19^.

As one of the important graphene derivatives, GO is a single sheet of sp^2^-hybridized carbon atoms arranged within a honeycomb lattice. It possesses many π-conjugated structure in the graphitic basal plan, which can form strong π-π stacking interaction with the π-conjugated system (such as benzene ring) in PEEK^20^,^21^,^22^. For example, Wu *et al*. has evaluated the adsorption characteristics of graphene toward methyl blue, p-toluenesulfonic acid as well as 1-naphthalenesulfonic acid, and found that the adsorption of these chemicals containing benzene rings on graphene was a π-π stacking adsorption process^20^. Besides, GO is highly negatively charged because of the presence of large amounts of oxygen functional groups (such as carboxylic acids as well as phenolic hydroxyls) on their surface and edges^23^,^24^,^25^. The negatively charged GO can absorb the positively charged calcium atoms (C sites) in the (100) crystal plane of HAP^26^,^27^ through electrostatic interactions. Our previous work had already demonstrated that GO could form strong interface bonding with positively charged bioceramic^28^. Therefore, GO can be used as an interface phase to combine the biopolymer and bioceramic.

Most recently, GO has been used to absorb some organic chemicals containing benzene rings or pyridine such as polystyrene^29^, polyaniline^30^, DNA^31^, and porphyrin^32^ through π-π stacking interaction, or to absorb some inorganic particles containing cations such as Ag_3_PO_4_^33^, ZnO^34^ and Al_2_O_3_^35^ through electrostatic interaction. However, few study has used GO as interface phase to absorb organic chemicals and inorganic particles simultaneously. Apart from the unique interfacial characteristics, GO also has excellent Young’s modulus (~1.0 TPa) as well as ultimate breaking strength (~130 GPa) which can greatly improve the mechanical properties^36^,^37^. Rich surface oxygen functional groups (carboxyl as well as hydroxyl groups) imbue it with good hydrophilicity and surface activity that are benefit for cellular adhesion and proliferation, and apatite forming ability^38^,^39^,^40^,^41^.

Considering these issues, HAP was incorporated into PEEK to improve the bioactivity and bone bonding ability. Meanwhile, GO were used to improve the interfacial bonding and compatibility between HAP and PEEK. The PEEK-HAP/GO scaffolds were fabricated via selective laser sintering (SLS). The phase compositions and morphologies of the scaffolds were determined using X-ray diffraction (XRD) and scanning electron microscopy (SEM), respectively. Thermal characterizations were performed by thermogravimetric analysis (TGA) and differential scanning calorimetry (DSC). The determination of the compressive strength and modulus was performed by compressive tests for a wide range of GO content. The bioactivity, cytocompatibility and biocompatibility were investigated via *in vitro* and *in vivo* experiments.

## Results

**Figure 1:**
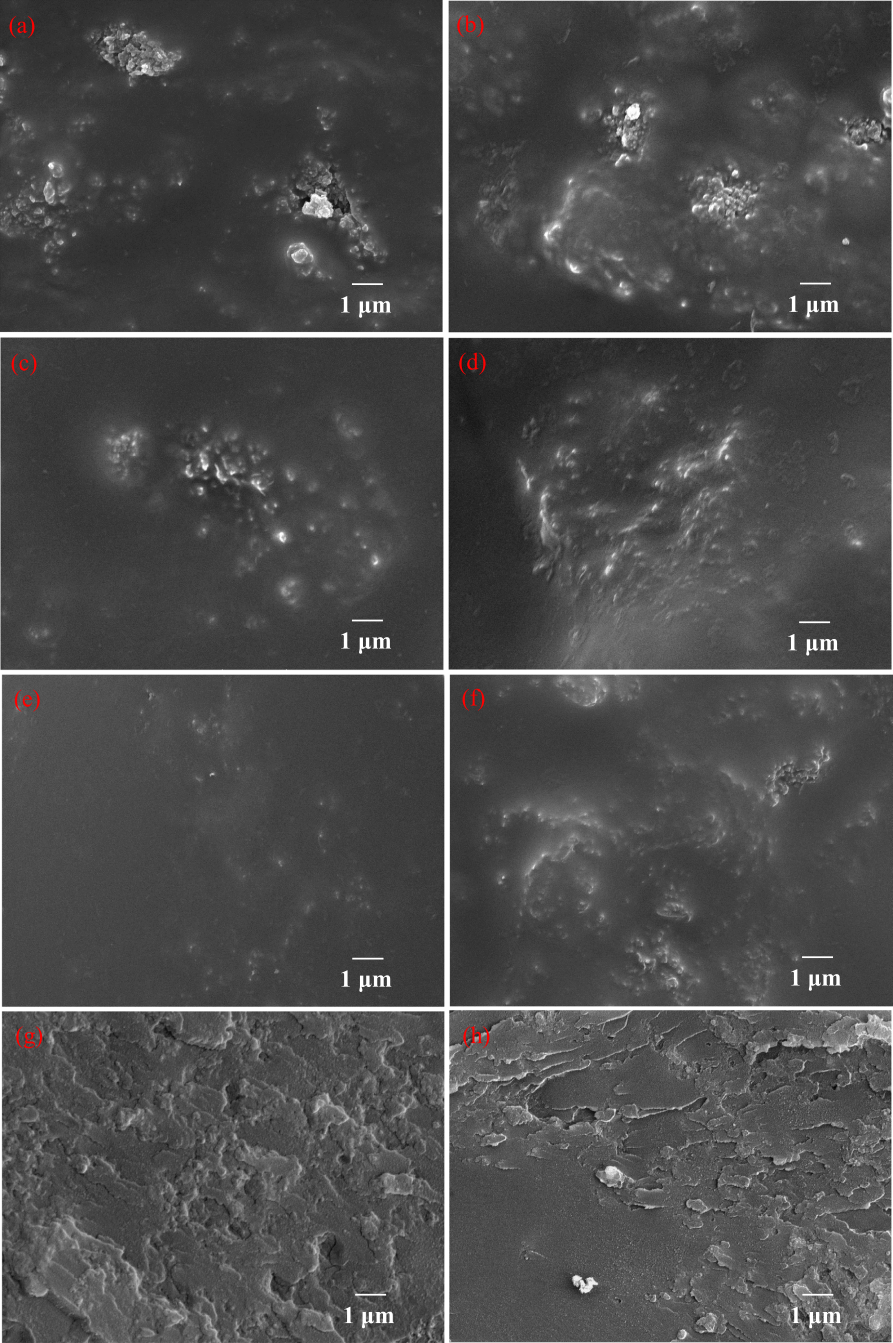
SEM images. Surface of (**a**) PEEK-HAP scaffolds, (**b**–**f**) PEEK-HAP/GO scaffolds with (**b**) 0.25 wt%, (**c**) 0.5 wt%, (**d**) 0.75 wt%, (**e**) 1 wt% and (**f**) 1.25 wt% GO, and (**g**,**h**) fracture surface of PEEK-HAP/GO scaffolds with (**g**) 1 wt% and (**h**) 1.25 wt% GO.

The improvement of interfacial bonding strength can be reflected by the dispersibility and compatibility. The surface morphologies of the PEEK-HAP/GO scaffolds with different GO content are presented in Fig. 1, and the PEEK-HAP scaffolds were used as control. For the PEEK-HAP scaffolds, nano-HAP particles were non-uniformly distributed and agglomerated in the PEEK matrix (Fig. 1a). Similar results have been published by others about biopolymer-bioceramic based composites, which were explained by the poor compatibility due to the great differences in the physical and chemical properties between biopolymer and bioceramic, leading to a low interfacial contact with the PEEK matrix^42^. For the PEEK-HAP/GO scaffolds, the degree of agglomeration decreased with the increasing of GO content (Fig. 1b–e). While the dispersibility became poor and some agglomerated nano-HAP particles could easily observed (Fig. 1f) when the GO content was 1.25%. In the fracture surface of PEEK-HAP/GO scaffolds with 1 wt% GO, GO was uniformly distributed in the matrix, which might be beneficial for improving the interfacial bonding between HAP and PEEK (Fig. 1g). In contrast, the presence of GO agglomerates led to the nano-HAP agglomeration in the PEEK matrix, and some pores were observed in the PEEK-HAP/GO scaffolds with 1.25% GO (Fig. 1h).

**Figure 2:**
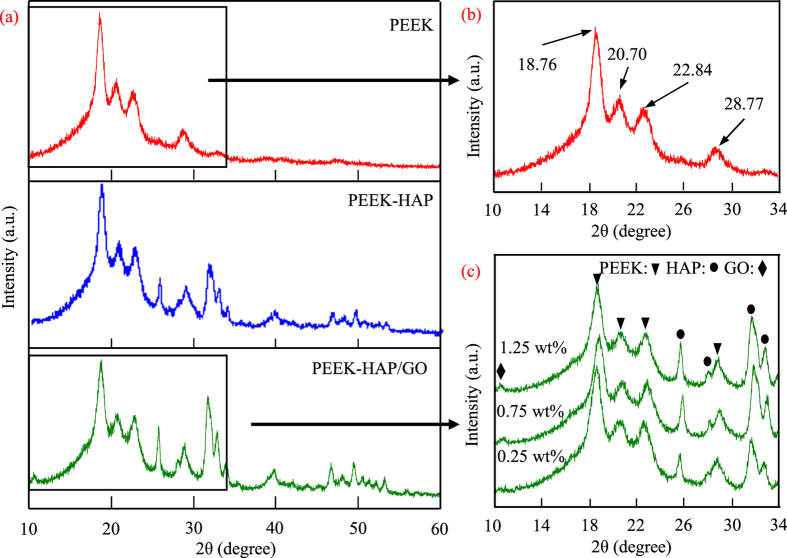
XRD patterns. (**a**) PEEK-HAP/GO, PEEK and PEEK-HAP scaffolds, (**b**) zoom in the 10–34° region for PEEK scaffolds, (**c**) zoom in the 10–34° region for PEEK-HAP/GO scaffolds with 0.25, 0.75 and 1.25 wt% GO.

The phase composition of the PEEK-HAP/GO scaffolds were characterized by XRD with the PEEK and PEEK-HAP scaffolds serving as control, as shown in Fig. 2. The PEEK scaffolds had four distinct peaks at 18.76°, 20.70°, 22.84° and 28.77°, corresponding to the (110), (111), (200) and (211) plane of semicrystalline PEEK, respectively (Fig. 2b). It was in agreement with the data reported previously^43^,^44^. The main diffraction peaks of HAP appeared at 25.7°, 31.8°, 33.8° and 39.8°, corresponding to the diffraction planes (002), (211), (202) and (300), respectively (Fig. 2a). The chemical composition of PEEK-HAP scaffolds was PEEK and HAP. The XRD pattern of PEEK-HAP/GO scaffolds had the same profile as observed in the PEEK-HAP scaffolds (Fig. 2c). While, the characteristic peak corresponding to GO was present at 10.80°, and the intensity of this peak was very low which might be possibly attributed to low weight fraction of GO. Furthermore, the intensity of this peak in the patterns increased with increasing amount of GO in the PEEK-HAP/GO scaffolds. The results indicated that the existence of HAP and GO in the PEEK matrix after laser sintering.

**Figure 3:**
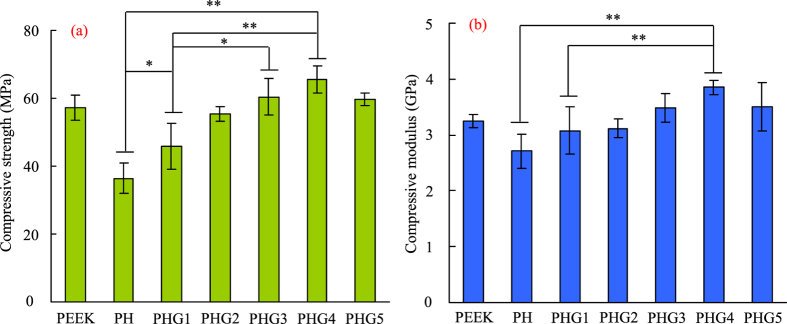
Compressive strength and modulus. Compressive strength (**a**) and modulus (**b**) of the PEEK-HAP/GO, PEEK and PEEK-HAP scaffolds with different GO content. The PEEK-HAP scaffolds and PEEK-HAP/GO scaffolds with 0.25 wt%, 0.5 wt%, 0.75 wt%, 1 wt%, 1.25 wt% GO were labeled PH scaffolds and PHG1, PHG2, PHG3, PHG4, PHG5, respectively. Data are presented as mean ± SD for n = 6 (^*^P < 0.05, ^**^P < 0.01).

The mechanical properties of the PEEK-HAP/GO scaffolds with different GO content were evaluated through compression test and the results are presented in Fig. 3. The PEEK-HAP scaffolds’ compressive strength and modulus were approximately 36.45 MPa and 2.71 GPa, respectively, while the compressive strength and modulus of the PEEK scaffolds were approximately 57.23 MPa and 3.25 GPa, respectively. The compressive strength increased initially to 65.41 MPa at 1 wt% GO, while decreased slightly at higher GO contents. And the compressive modulus of PEEK-HAP/GO0.25, PEEK-HAP/GO0.5, PEEK-HAP/GO0.75, PEEK-HAP/GO1 and PEEK-HAP/GO1.25 scaffolds increased by 13.65%, 15.13%, 28.41%, 42.07% and 29.52%, respectively, when compared with PEEK-HAP scaffolds.

**Figure 4:**
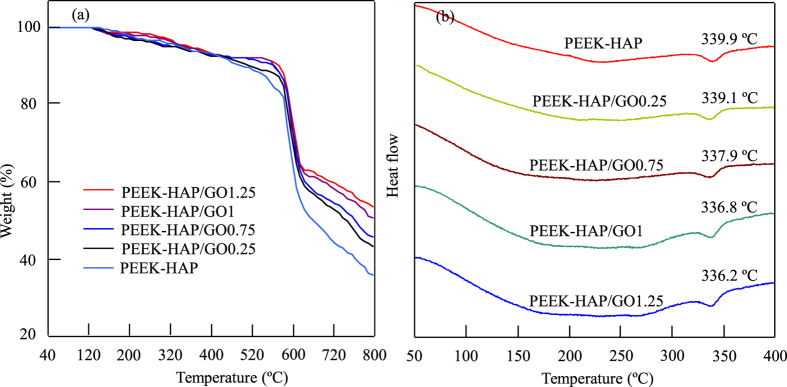
Thermal analysis. TGA curve (**a**) and DSC curve (**b**) of PEEK-HAP and PEEK-HAP/GO scaffolds with 0.25, 0.75, 1 and 1.25 wt% GO.

Thermal characterizations of the scaffolds were determined by TGA and DSC analyses (Fig. 4). The characteristic temperatures of the PEEK-HAP and PEEK-HAP/GO1 scaffolds were tabulated in Table 1. It could be seen that the PEEK-HAP/GO and PEEK-HAP scaffolds decomposed in a two steps process, and the TGA curve profile of the PEEK-HAP/GO scaffolds shifted to a higher temperature compared to that of the PEEK-HAP scaffolds (Fig. 4a). The onset temperature of degradation for the PEEK-HAP/GO scaffolds with 1 wt% GO loading was about 3 °C higher than that without GO loading. The temperatures at 5% weight loss were 357 °C and 353 °C for the PEEK-HAP/GO1 scaffolds and PEEK-HAP scaffolds, respectively. The decomposition temperature of the PEEK-HAP/GO1 scaffolds was 571 °C which was 9 °C higher than that of the PEEK-HAP scaffolds. The results indicated that the PEEK-HAP/GO1 scaffolds had a higher thermal stability than the PEEK-HAP scaffolds. The melting point of PEEK-HAP/GO0.25 scaffolds was 339.1 °C, only 0.8 °C lower than that of PEEK-HAP scaffolds in the DSC thermograms (Fig. 4b). The melting point of the PEEK-HAP/GO scaffolds decreased with increasing GO content. While the crystallinity of the PEEK-HAP and PEEK-HAP/GO scaffolds were almost kept the same.

**Figure 5:**
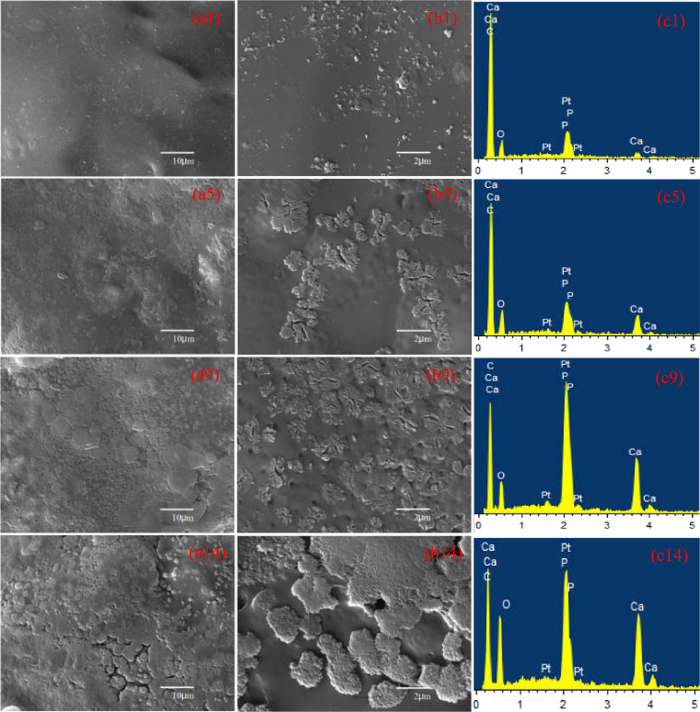
Apatite formation on the PEEK-HAP/GO1 scaffolds. 1 day (**a1**,**b1** and **c1**), 5 days (**a5**,**b5** and **c5**), 9 days (**a9**,**b9** and **c9**) and 14 days (**a14**,**b14** and **c14**): Low-magnification (**a1**,**a5**,**a9** and **a14**) and high-magnification (**b1**,**b5**,**b9** and **b14**) SEM images, and EDS spectra (**c1**,**c5**,**c9** and **c14**).

Scaffolds’ bioactivity was evaluated by immersing in SBF and then observed the formation of bone-like apatite phase. Apatite formed on the PEEK-HAP/GO1 scaffolds surface after SBF immersion for 1, 5, 9 and 14 days is shown in Fig. 5. Some apatite particles were deposited on the scaffolds’ surface after being soaked in SBF for 1 day (Fig. 5a). An increasing number of apatite particles were present, and the particles size increased as the soaking time increased (Fig. 5b and c). After 14 days, a newly formed apatite layer was observed, which was sponge-like and compact (Fig. 5d). The elements were mainly Ca, P, C and O according to the EDS spectra. The Ca/P ratio of apatite deposited on the scaffolds was increased from 1.22 to 1.63, as immersion time increased from 1 to 14 days. It was close to the Ca/P ratio in stoichiometric HAP (1.67), suggesting that bone-like apatite formed. Many studies have demonstrated that bone-like apatite plays a vital role in forming a chemical bond with bone tissue, and could enhance osteoconductivity^45^,^46^,^47^.

**Figure 6:**
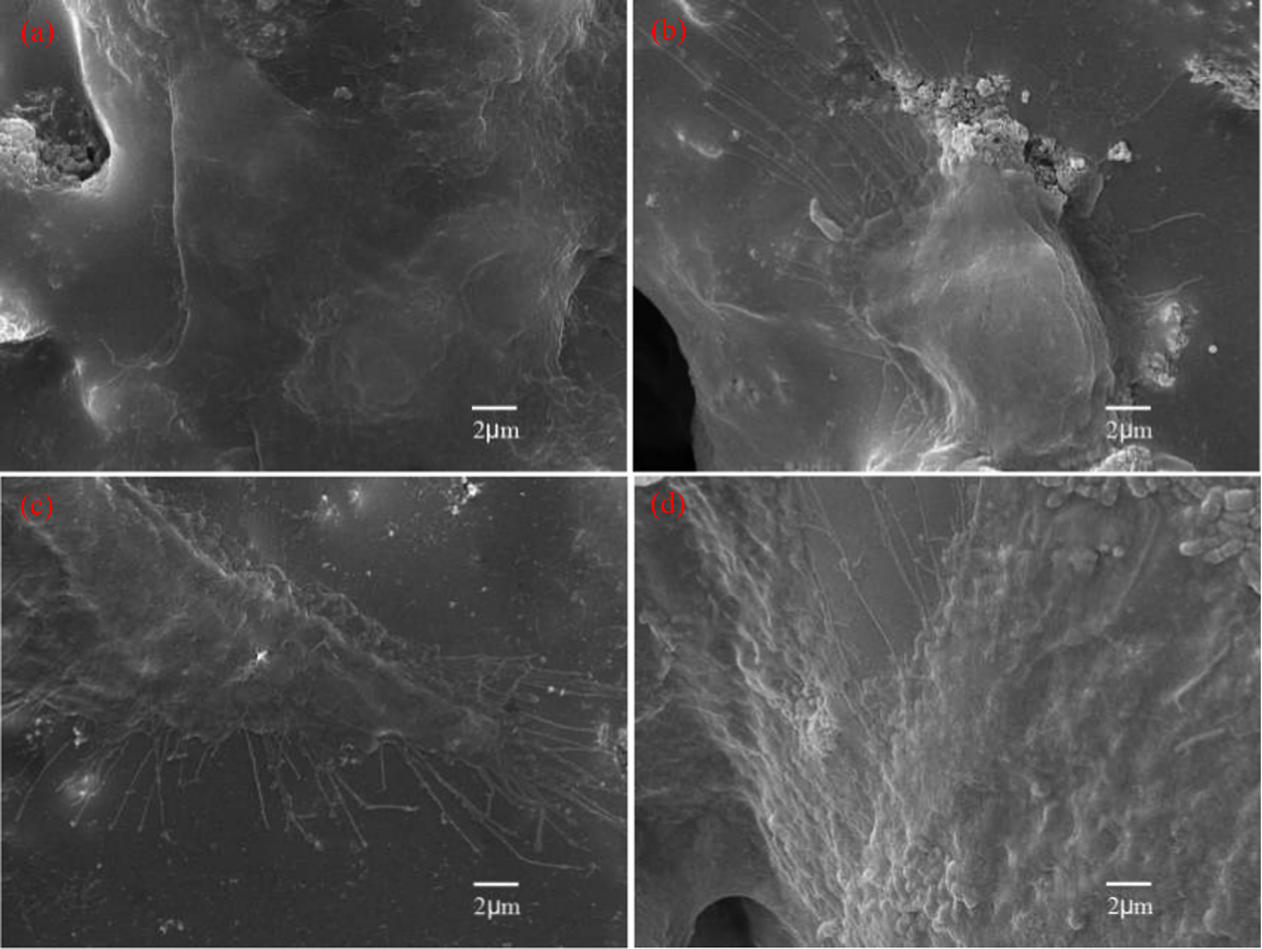
MG-63 cell attachment and proliferation on the PEEK-HAP/GO1 and PEEK-HAP scaffolds. SEM images of the attachment of MG-63 cells on the PEEK-HAP scaffolds (**a** and **b**) and PEEK-HAP/GO1 scaffolds (**c** and **d**) for 3 (**a** and **c**) and 7 days (**b** and **d**).

The cytocompatibility of scaffolds was evaluated using MG-63 cells, and the morphologies of cells adhered to the PEEK-HAP/GO1 and PEEK-HAP scaffolds for different time are shown in Fig. 6. It was observed that MG-63 cells could attach to the surface of the scaffolds. The cells covered almost the entire surface of the PEEK-HAP/GO1 scaffolds and approximately 40% of the PEEK-HAP scaffolds. The quantitative analysis results of the cells attachment and proliferation are shown in Figure S1. The cells adhesion on the PEEK-HAP/GO1 and PEEK-HAP scaffolds after 2 h incubation at 37 °C in a 5% CO2 humidified atmosphere incubator are shown in Figure S1a. The cells adhesion on the PEEK-HAP/GO1 scaffolds was significant higher than that on the PEEK-HAP scaffolds after 2 h of cell incubation. The cells proliferation on the PEEK-HAP/GO1 and PEEK-HAP scaffolds for 1, 3, 5 and 7 days are presented in Figure S1b. It could be seen that both the scaffolds could support MG-63 cell proliferation. No significant differences of cell number appeared on day 1, and the cell number increased with increasing of culture time for both the scaffolds.

**Figure 7:**
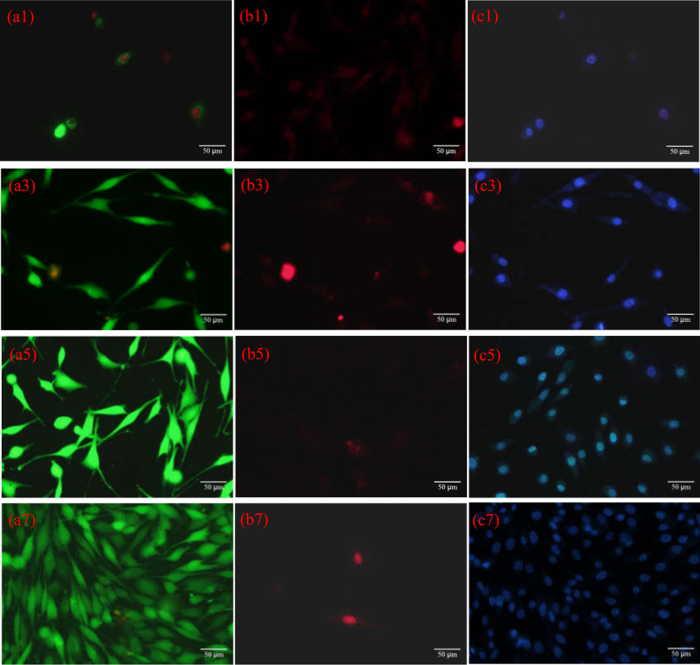
Fluorescent images. Live cells (green, **a1**,**a3**,**a5**,**a7**), dead cells (red, **b1**,**b3**,**b5**,**b7**) and cell nuclei (blue, **c1**,**c3**,**c5**,**c7**) through staining after cell culture on the PEEK-HAP/GO1 scaffolds for 1 day (**a1**,**b1** and **c1**), 3 days (**a3**,**b3** and **c3**), 5 days (**a5**,**b5** and **c5**) and 7 days (**a7**,**b7** and **c7**).

Fluorescent images of live cells, dead cells and cell nuclei through staining after cell culture on the PEEK-HAP/GO1 scaffolds for different time points are shown in Fig. 7. The live MG-63 cells and cell nuclei were stained green and blue, respectively. They exhibited the classical fusiform shape, suggesting normal cell growth. The density of cells attached to the scaffolds greatly increased from 3 to 7 days. The dead MG-63 cells were stained red, and there were very few dead cells on the scaffolds at all time points. This demonstrated that the PEEK-HAP/GO1 scaffolds offered good support for cells. ALP staining images of the cells after culturing on the PEEK-HAP/GO1 scaffolds at different times are shown in Figure S2. MG-63 cells could be identified by the strong positive staining reaction for ALP.

**Figure 8:**
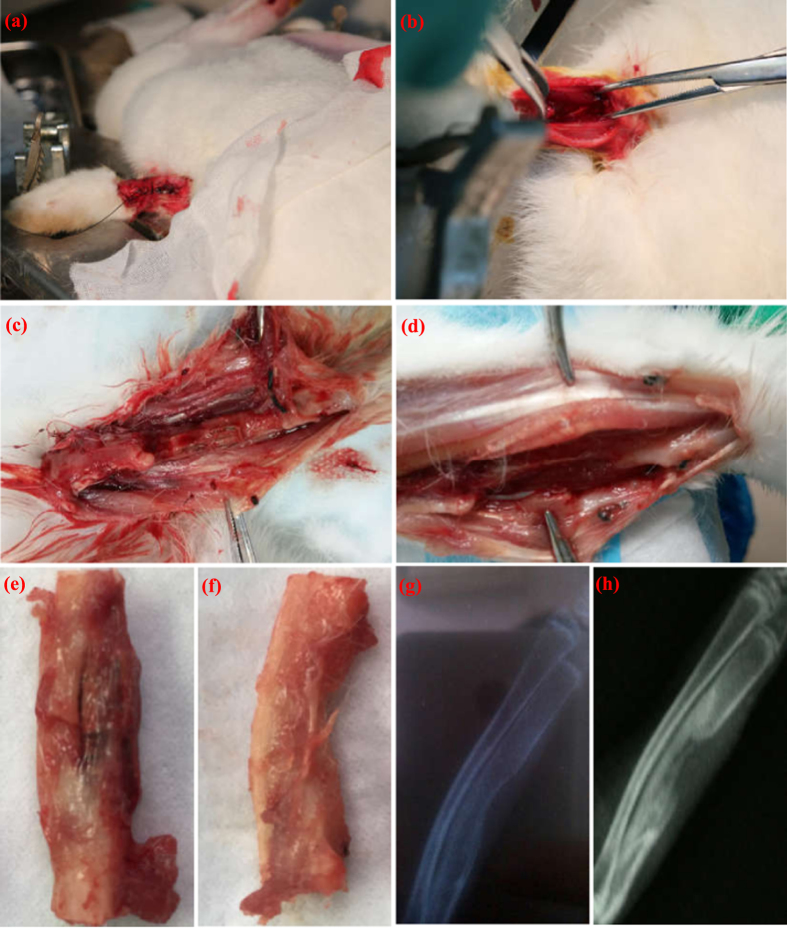
Bone defects created in the rabbit radius and X-ray images. Bone defects created in the rabbit radius with (**a** and **c**) and without the PEEK-HAP/GO1 scaffolds (**b** and **d**), specimens harvested at 60 days of implantation with (**e**) and without the PEEK-HAP/GO1 scaffolds (**f**), X-ray images of radius restoration after 60 days (**g** and **h**) implantation of the PEEK-HAP/GO1 scaffolds (**h**) and the control group (**g**).

To investigate the *in vivo* biocompatibility and bone regeneration ability, the scaffolds were implanted into the bone defect site of rabbits. The photographs of an established bone defect mold are shown in Fig. 8(a–d). The bone defect created in the right radius was packed with the PEEK-HAP/GO1 scaffolds (Fig. 8a and c), and the left radius served as a control (Fig. 8b and d). The incised skin healed and closed completely two days after surgery. The scaffolds were observed in the harvested specimens after 60 days of implantation (Fig. 8e). New bone formed along the scaffold surface and grew across the defect area. The rabbits were radiographed after surgery for 60 days, and the results are shown in Fig. 8(g,h). New bone was formed and covered almost all of the defect sites after 60 days of implantation (Fig. 8g). However, part of blank defect was still in control group (Fig. 8h).

## Discussion

**Figure 9:**
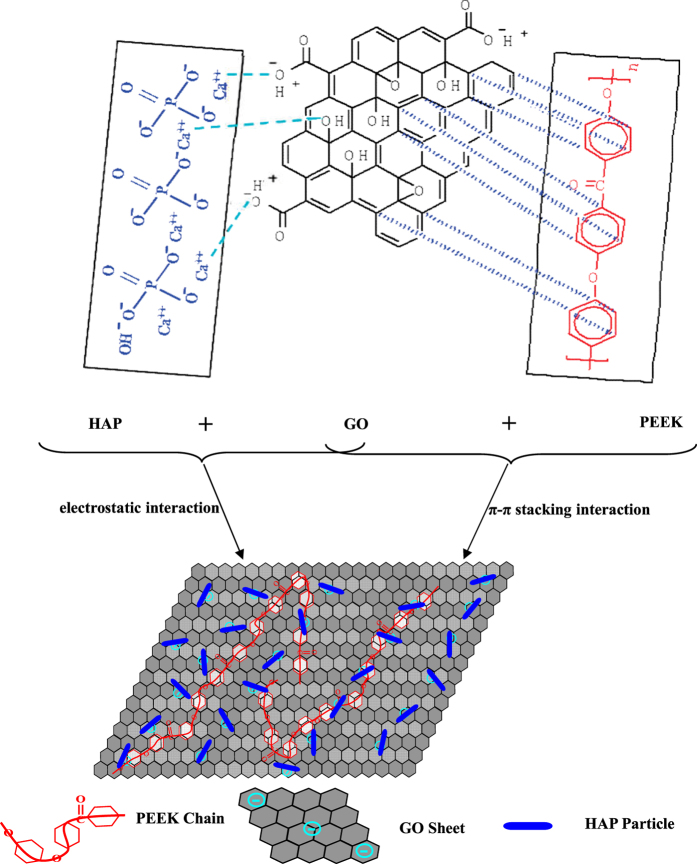
Schematic illustration of GO as an interface phase to combine PEEK and HAP.

The schematic illustration of GO promoting HAP dispersion as an interface phase is shown in Fig. 9. GO possessed large π-conjugated structure in the graphitic basal plan, and PEEK had π-conjugated system (benzene ring) spread along the backbone. The PEEK chains could be strongly adsorbed onto the surface of GO via π-π stacking interaction. Besides, GO was negatively charged because of the rich oxygen functional groups (such as carboxylic acids and phenolic hydroxyls) originating from the preparation procedure of the exfoliated graphites. While the (100) crystal plane of HAP was positively charged due to the present of calcium cations. After compositing, the positively charged calcium cations would be adsorbed on the negatively charged GO surface through electrostatic interaction. Therefore, with the role of GO, HAP was homogeneously dispersing in the PEEK matrix, and the compatibility between HAP and PEEK was improved.

As we know, scaffolds should have suitable mechanical strength to provide initial mechanical stability and support for cell attachment. The compressive strength and modulus of the PEEK-HAP scaffolds were approximately 36.45 MPa and 2.71 GPa, respectively, which were much lower than that of the PEEK scaffolds. Increasing GO content in the matrix increased the strength and modulus of the scaffolds, which implied that GO helped to improve the PEEK-HAP/GO scaffolds’ mechanical properties. The compressive strength and modulus increased initially to 65.41 MPa and 3.85 GPa at 1 wt% GO, while decreased slightly at higher GO contents. The mechanical properties of the PEEK-HAP/GO1 scaffolds were much higher than that of the PEEK-HAP scaffolds. The strength of the PEEK-HAP/GO1 scaffolds reached up to 65.41 MPa and 3.85 GPa, respectively, quantities comparable to human trabecular bone (strength: 0.1–16 MPa, modulus: 0.05–0.5 GPa)^48^,^49^.

TGA curve profile of the PEEK-HAP/GO scaffolds shifted to a higher temperature compared to that of the PEEK-HAP scaffolds. It indicated that the mobility of the PEEK polymer chain at the interfaces of PEEK and GO was suppressed by strong interaction, which improved the thermal stability^50^,^51^. The improved thermal stability in PEEK-HAP/GO scaffolds was due to an interaction between the π-conjugated structure of GO and the benzene ring of PEEK through π-π stacking interaction. The melting point of the PEEK-HAP/GO scaffolds decreased with increasing GO content. While the crystallinity of the PEEK-HAP and PEEK-HAP/GO scaffolds were almost kept the same. Previous studies had demonstrated that PEEK was a semicrystalline polymer, and the mechanical properties were strongly depended on the crystallinity^52^,^53^,^54^. While the crystallinity of PEEK changed very slight that could not produce great influence on mechanical properties. Therefore, the mechanical properties improvement might be ascribed to the evenly dispersion of GO and the strong interfacial interactions between GO and matrix. GO could form good interfacial bonding with PEEK and HAP through π-π stacking interaction and electrostatic interaction, respectively. The strong interface could transfer stress directly from matrix to GO, thus improving the mechanical properties. As the PEEK-HAP/GO1 scaffolds had the optimal mechanical properties for bone tissue engineering, we used the PEEK-HAP/GO1 scaffolds to carry out the biological properties tests, and the PEEK-HAP scaffolds were used as control.

Ideal scaffolds for bone tissue engineering application must also possess good biocompatibility with cells and tissues. Cells cultured on the PEEK-HAP/GO1 scaffolds exhibited many protrusive fine filopodia (microspikes), indicating that MG-63 cells preferred the PEEK-HAP/GO1 scaffolds over the PEEK-HAP scaffolds. The PEEK-HAP/GO1 scaffolds had a significantly higher cell adhesion number than the PEEK-HAP scaffolds after 3 days (*P* < 0.05), indicating that the PEEK-HAP/GO1 scaffolds contributed more to improve the activity of the MG-63 cells than the PEEK-HAP scaffolds due to the addition of GO. Several researchers have previously reported that the addition of GO to the biopolymer matrix could enhance cell attachment and proliferation^55^,^56^,^57^. The ALP staining tests results demonstrated that the PEEK-HAP/GO1 scaffolds provided a favorable microenvironment for MG-63 cells osteogenic differentiation. Previous studies have shown that GO had a tremendous effect on the behavior of osteoblasts and stem cells because the oxygen functional groups could produce a negatively charged surface^58^,^59^,^60^. The negatively charged surface was good for hydrogen bonding and the electrostatic force interactions between the GO and cells as well as specific proteins absorption.

**Figure 10:**
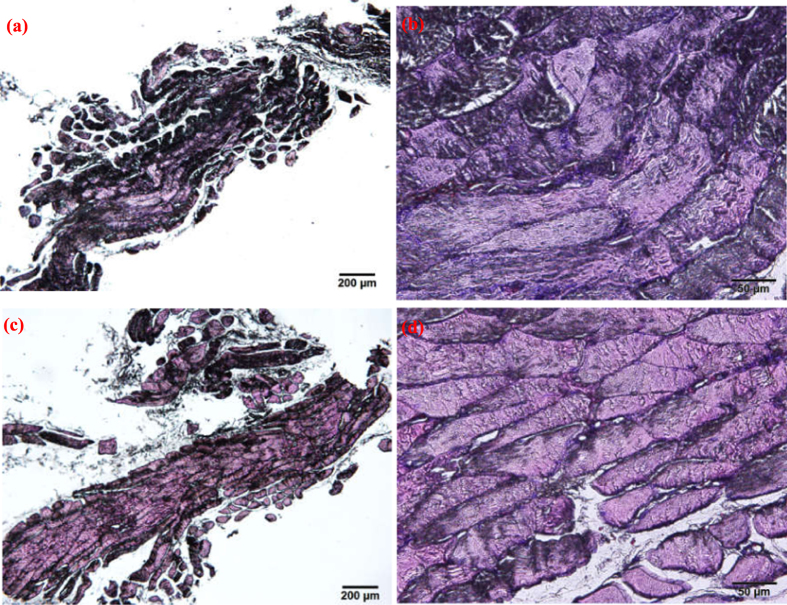
Histological vertical sections. Autologous bone tissue (**a** and **b**) and the scaffold with surrounding tissue (**c** and **d**) at 60 days postimplantation with two magnifications, (**a** and **c**) 4× and (**b** and **d**) 20×.

Histological vertical sections of the scaffold with surrounding tissue and autologous bone tissue at 60 days postimplantation are presented in Fig. 10. No significant differences were observed in tissue formation between the two groups. It could be seen that the scaffold had been replaced with spongy bone tissue, including a large number of trabecular bone tissues, fibrous tissue and vascular tissue. Some cavities formed because of the scaffold degradation, which was essential to provide a benefit space for vascular tissue ingrowth followed by new bone tissue regeneration. It demonstrated that the PEEK-HAP/GO1 scaffolds had good efficacy for repairing bone defects. The results were in agreement with previous studies showing that the bone-like apatite layer produced on scaffolds surface could enhance bone regeneration due to the addition of bioceramic to the biopolymer matrix^61^,^62^.

Previous studies have showed that the properties of GO-based materials depend on the sizes, compositions and structures of the GO sheets^63^,^64^,^65^. For example, small GO sheets have good dispersibility and biocompatibility, while lager GO sheets have better mechanical properties. In this paper, GO had a diameter of 1–5 μm and a thickness of 0.8–1.2 nm. It had excellent modulus and strength which can greatly improve the mechanical properties of scaffolds. The strength and modulus of the PEEK-HAP scaffolds with 1 wt% GO were increased by 180% and 142%, respectively. More importantly, GO was highly negatively charged because of the presence of large amounts of oxygen functional groups (such as carboxylic acids as well as phenolic hydroxyls) on their surface and edges. And it also possessed π-conjugated structure in the graphitic basal plan. Therefore, GO could absorb HAP and PEEK through electrostatic interactions and π-π stacking interaction, respectively. In addition, rich surface oxygen functional groups imbued GO with good biocompatibility, hydrophilicity and surface activity. The *in vitro* and *in vivo* experiments showed that GO could accelerate apatite forming, cellular adhesion, proliferation and differentiation, and bone tissue regeneration.

## Conclusions

In this study, the PEEK-HAP/GO scaffolds with interconnected pore network were fabricated via SLS. The interface bonding strength between HAP and PEEK increased with increasing GO content from 0 to 1 wt%, but decreased as more GO was added. The increased interface bonding strength was due to the uniform dispersion of GO which served as an interface phase to combine HAP and PEEK, while the decreased was due to the GO agglomeration. Bone-like apatite layer grew uniformly on the entire surface of the scaffold after SBF incubation, indicating the good bioactivity. The cell seeding, attachment, proliferation and differentiation on the PEEK-HAP/GO scaffolds were markedly better than those on the PEEK-HAP scaffolds. The *in vivo* experiments based on bone defect repair confirmed that the scaffolds possessed excellent biocompatibility and highly efficient for guiding new bone formation. The PEEK-HAP/GO scaffolds with good mechanical and biological properties have the potential for use in tissue regeneration.

## Materials and Methods

### Materials and reagents

A commercially available PEEK powder (Dongguan Guanhui Plastic Materials Co. Ltd., Guangdong, China) with mean particle sizes of about 20 μm was used as received. HAP nanopowder (Nanjing Emperor Nano Material Co. Ltd., Jiangsu, China) was prepared by wet precipitation method with 150 nm length and 20 nm width, and characterized as described previously^66^. GO (Nanjing Jcnano Technology Co. Ltd., Jiangsu, China) was synthesized from graphite powder by a modified Hummer’s method with a thickness of 0.8–1.2 nm and a diameter of 1–5 μm.

Dulbecco’s phosphate-buffered saline (PBS), Dulbecco’s modified Eagle’s medium (DMEM) and fetal bovine serum (FBS) were obtained from Cellgro-Mediatech Inc. (Manassas, VA, USA). 4,6-diamidino-2-phenylindole (DAPI), α-minimal essential medium (α-MEM), dimethyl sulfoxide (DMSO), trypsin ethylenediaminetetraacetic acid (EDTA), (3-(4,5-Dimethylthiazol-2-yl)-2,5-diphenyltetrazolium bromide) (MTT), calcein-AM and propidium iodide (PI) were purchased from Sigma-Aldrich (St. Louis, MO, USA) and used as received. All other reagents were bought from Life Technologies (Eggenstein, Germany).

### Fabrication of scaffolds

The PEEK-HAP/GO composite powder with 1 wt% GO was used as an example to describe the preparation procedure. Firstly, PEEK powder (1.6 g) and HAP powder (0.4 g) were added to a beaker containing 20 ml of distilled water, and treated with sonicating for 30 min at 50 °C and magnetic stirring for another 30 min. GO (0.1 g) was added to another beaker containing 20 ml of distilled water, and also treated with sonicating for 30 min at 50 °C and stirring for another 30 min. Then GO aqueous suspension was gradually dropped into the PEEK-HAP solution and a homogeneous PEEK-HAP/GO solution was obtained after ultrasonic treatment at 50 °C for 1 h and stirring for 1 h. Finally, the suspensions were filtered through a fresh membrane at room temperature and the filtration residues were dried in an electrothermal blowing dry box (101-00S, Guangzhou Dayang Electronic Machinery Equipment Co., Ltd, China) at 80 °C for 12 h. Other types of composite powder with different GO content were fabricated according to the same procedures.

A thin layer (thickness: 0.1–0.2 mm) of composite powder was paved evenly on the powder bed. Scaffolds were fabricated by selectively sintering the powder with a laser beam layer by layer. The SLS processing was conducted by scanning the laser at 2.8 W power, 1.0 mm spot diameter and 180 mm/min scanning speed. The fabrication procedure of the PEEK-HAP/GO scaffolds is shown in Figure S3. The PEEK-HAP scaffolds with GO was labeled PEEK-HAP/GO scaffolds, and the PEEK-HAP scaffolds with 0.25, 0.5, 0.75, 1, 1.25 wt% of GO were labeled PEEK-HAP/GO0.25, PEEK-HAP/GO0.5, PEEK-HAP/GO0.75, PEEK-HAP/GO1, PEEK-HAP/GO1.25, respectively. The morphologies of the scaffolds (10 mm × 10 mm × 5 mm) under the optical microscope are given and they exhibit interconnecting pore structure.

### Characterization

The morphologies of the scaffolds, mineralization deposition on scaffold surface after simulated body fluid (SBF) soaking, and MG-63 cell adhesion on scaffold after cell culturing were examined by a SEM (FEI Quanta-200, FEI Co., USA). All samples were sputtered with gold using an auto fine coater (JFC-1600, JEOL, Ltd., Japan) for 30 s at an accelerating voltage of 8 kV. The phase compositions of the scaffolds were analyzed using XRD (D8 Advance, German Bruker Co., German). The XRD patterns were recorded between 10° to 60° (2 θ) in step of 8 intervals with a 1 min counting time at each step and operating parameters were 40 kV and 40 mA with CuKα monochromatic radiation.

The compressive strength and modulus of the scaffolds (10 mm × 10 mm × 5 mm) were tested using a universal testing machine (WD-D1, Shanghai Zhuoji Instruments Co. LTD, China) at a cross-head speed of 0.5 mm/min. Six samples were used for replicating the experiment. The thermal properties were studied by TGA and DSC with 10 °C/min heating rate using a synchronous thermal analyzer (STA-200, Nanjing Dazhan institute of electromechanical technology, China). Approximately 8 mg sample was taken in an aluminum crucible under a nitrogen flow (100 ml/min). TGA and DSC were conducted from 40 to 800 °C and 50 to 400 °C, respectively. The thermal degradation temperature taking into account were the temperature at onset (T_onset_), the temperature at 5% weight loss (T_5_) and decomposition temperatures (T_d_). T_d_ was defined as the intersection of the baseline before decomposition and the tangent to the mass loss afterward.

### Mineralization study in SBF

To evaluate the mineralization of scaffolds, SBF contained similar composition and concentrations to those of human blood plasma, which was prepared as previously reported^67^. The scaffold samples (10 mm × 10 mm × 5 mm) were immersed in 200 ml of SBF at 37 °C. They were removed from SBF at predetermined time intervals of 1, 5, 9 and 14 days, gently rinsed with distilled water, left to dry at 37 °C for 1 day and characterized by SEM and energy dispersive spectroscopy (EDS, Neptune XM4, EDAX Inc., USA).

### Cells adhesion, proliferation and osteogenic differentiation

MG-63 human osteoblast-like cells were cultured in DMEM containing 10% FBS and 1% penicillin-streptomycin. They were maintained in a humidified 5% CO_2_ incubator at 37 °C, and the culture medium was refreshed every second day. MG-63 cells were used to evaluate the interaction of cells with PEEK-HAP/GO1 scaffolds, including adhesion, proliferation as well as differentiation. And the PEEK-HAP scaffolds were used as control. Before cells were seeded, the scaffold samples (10 mm × 10 mm × 5 mm) were sterilized with 70% ethanol for 30 min, and then washed three times with PBS. Subsequently, they were transferred to fresh culture dishes and the cells were seeded onto the scaffolds at a density of 4 × 10^5^ cells per sample.

To evaluate cell attachment, the cell/scaffold constructs were washed twice with PBS and immobilized with 3% glutaraldehyde for 2 h. The additional glutaraldehyde was removed by rinsing with distilled water, followed by dehydration with upgrading concentrations of ethanol (from 30% to 100%). Samples were then allowed to dry in the dry box for 12 h, sputtered with gold, and then examined using SEM. The number of living cells was measured according to MTT-based colorimetric assays. This assay relies upon the ability of living cells to reduce a tertrazolium salt into soluble coloured formazan product. After 2 h of cell incubation in a humidified 5% CO_2_ incubator at 37 °C, non-adherent cells were removed by two gentle washes in PBS. 2 ml MTT solution (5 mg/ml in PBS) was added to each well and cultured at 37 °C in a humidified atmosphere for 4 h. Then the supernatant was removed carefully and formazan product was dissolved by adding 300 ml DMSO. 100 μl of solution was transferred to a fresh 24-well plate. The absorbance at λ = 570 nm was determined using a microplate reader. The quantitative analysis of MG-63 cells proliferation on the scaffolds was assessed by the MTT assay as described above. Cells were incubated on the different substrates for 1, 3, 5 and 7 days before analysis. Results were reported as optical density units. Six samples were tested for each incubation period and each test was performed in triplicate.

For cell viability studies, MG-63 cells were seeded on the scaffolds at a density of 1 × 10^5^ cells per sample and cultured at 37 °C in a 5% CO_2_ incubator. After different culturing time, the cell/scaffold constructs were washed with PBS and exposed to 2 μM calcein-AM and 4 μM PI for 40 min. Nuclei were stained with DAPI at a concentration of 8 μM. The stained scaffolds were analysed using a fluorescence microscope (Olympus Co. Ltd., Tokyo, Japan) equipped with a digital camera (Olympus America Inc., Mel-ville, NY, USA) to visualize the living and dead cells. Stained images were obtained in which green color, red color and blue color indicated live cells, dead cells and cell nuclei, respectively. The alkaline phosphatase (ALP) activity of MG-63 cells was assessed using both quantitative and qualitative assays. It was determined by a LabAssay^TM^ ALP kit (Wako, Osaka, Japan). At each time point, aliquots of 400 μl of 0.1% Triton X-100 solution were added to each well, and then the mixture was cultured at 4 °C for 2 h. The ALP activity of the lysates was determined using p-nitrophenyl phosphate as a substrate, and the absorbance was measured at 405 nm with a spectrophotometer. After washing in distilled water, the scaffolds were imaged by a light microscopy.

### *In vivo* osteointegration evaluation

All handling and surgical procedures were approved by the Institutional Animal Experiment Committee of the Xiangya Hospital, Central South University, China. The methods were carried out in accordance with the approved guidelines. Male New Zealand white rabbits (weight, 2.0–2.5 kg; age, 4 months) were used in the animal tests. Each rabbit had been anesthetized by intramuscular injection using l ml/kg of 3% pentobarbital sodium prior to surgery, and the surgery was performed under sterile conditions. A 20 mm incision was created in the middle shaft of the right radius. The subcutaneous tissues were separated from the periosteum to expose the radial diaphysis, and a 10 mm segmental bone defect was made. The PEEK-HAP/GO1 scaffolds were sterilized with ethylene oxide steam before use and were then placed in the defect sites. The defects of the left radius were left empty as a control. When the surgery implantation was done, the incisions were closed using absorbable sutures. All the rabbits received antimicrobial therapy for 3 days.

To evaluate new bone regeneration in bone defect, the rabbits were radiographed at 60 days using X-ray equipment (Siemens Heliodent, Erlangen, Germany) at an exposure of 5 s and energy of 50 kV. The degree of new bone formation was estimated using the grey scale which was displayed automatically by the digital X-ray imaging system. For histological study, the rabbits were sacrificed at 60 days after surgery. The implants were harvested with surrounding tissue, washed with normal saline, fixed by 10% neutral buffered formaldehyde, decalcified by ethylene diamine tetraacetic acid (EDTA), and embedded in paraffin. Vertical sections with 3–5 μm thickness were made and stained with hematoxylin & eosin (HE). The area of newly formed bone was observed under an optical microscopy.

### Statistical analysis

The data from compression tests and MTT assays were reported as the mean ± standard deviation (SD). All analyses were performed using SPSS (IBM Co., USA), and statistical comparisons were determined by the Student’s *t*-test. A *P* value of less than 0.05 was considered to indicate statistical significance.

## Additional Information

**How to cite this article:** Peng, S. *et al*. Graphene oxide as an interface phase between polyetheretherketone and hydroxyapatite for tissue engineering scaffolds. *Sci. Rep.* **7**, 46604; doi: 10.1038/srep46604 (2017).

**Publisher's note:** Springer Nature remains neutral with regard to jurisdictional claims in published maps and institutional affiliations.

## Supplementary Material

Supporting Information

## Figures and Tables

**Table 1:** Characteristic temperatures of the PEEK-HAP and PEEK-HAP/GO1 scaffolds.

Scaffold	T_onset_/°C	T_5_/°C	T_d_/°C
PEEK-HAP	148	353	562
PEEK-HAP/GO1	151	357	571

## References

[b1] Elschner C. . *In vitro* response of human mesenchymal stromal cells to titanium coated peek films and their suitability for magnetic resonance imaging. J. Mater. Sci. Technol. 31, 427–436 (2015).

[b2] Lee J. H. . *In vitro* and *in vivo* evaluation of the bioactivity of hydroxyapatite-coated polyetheretherketone biocomposites created by cold spray technology. Acta. Biomater. 9, 6177–6187 (2013). 23212079 10.1016/j.actbio.2012.11.030

[b3] Edwards S. L. & Werkmeister J. A. Mechanical evaluation and cell response of woven polyetheretherketone scaffolds. J. Biomed. Mater. Res. A. 100, 3326–3331 (2012). 22733655 10.1002/jbm.a.34286

[b4] Zhao Y. . Cytocompatibility, osseointegration, and bioactivity of three-dimensional porous and nanostructured network on polyetheretherketone. Biomaterials. 34, 9264–9277 (2013). 24041423 10.1016/j.biomaterials.2013.08.071

[b5] Tourani H. . Effects of fibers and nanoparticles reinforcements on the mechanical and biological properties of hybrid composite polyetheretherketone/short carbon fiber/Nano-SiO_2_. Polyme. Composite. 34, 1961–1969 (2013).

[b6] Fu S. Z. . *In vivo* biocompatibility and osteogenesis of electrospun poly (ε-caprolactone)-poly (ethylene glycol)-poly (ε-caprolactone)/nano-hydroxyapatite composite scaffold. Biomaterials. 33, 8363–8371 (2012). 22921926 10.1016/j.biomaterials.2012.08.023

[b7] Feng P., Niu M., Gao C., Peng S. & Shuai C. A novel two-step sintering for nano-hydroxyapatite scaffolds for bone tissue engineering. Sci. Rep-UK 4, 5599 (2014). 10.1038/srep05599 PMC4083286 24998362

[b8] Zhou H. & Lee J. Nanoscale hydroxyapatite particles for bone tissue engineering. Acta Biomater. 7, 2769–2781 (2011). 21440094 10.1016/j.actbio.2011.03.019

[b9] Núñez J. D. . Integration and bioactivity of hydroxyapatite grown on carbon nanotubes and graphene oxide. Carbon 79, 590–604 (2014).

[b10] Wang M. C. . Crystalline size, microstructure and biocompatibility of hydroxyapatite nanopowders by hydrolysis of calcium hydrogen phosphate dehydrate (DCPD). Ceram. Int. 41, 2999–3008 (2015).

[b11] Bose S., Roy M. & Bandyopadhyay A. Recent advances in bone tissue engineering scaffolds. Trends Biotechnol. 30, 546–554 (2012). 22939815 10.1016/j.tibtech.2012.07.005 PMC3448860

[b12] Jiang H. . Biomimetic spiral-cylindrical scaffold based on hybrid chitosan/cellulose/nano-hydroxyapatite membrane for bone regeneration. ACS Appl. Mater. Inter. 5, 12036–12044 (2013). 10.1021/am4038432 24191736

[b13] Gentile P., Chiono V., Carmagnola I. & Hatton P. V. An overview of poly (lactic-co-glycolic) acid (PLGA)-based biomaterials for bone tissue engineering. Int. J. Mol. Sci. 15, 3640–3659 (2014). 24590126 10.3390/ijms15033640 PMC3975359

[b14] Fernandez J. M., Molinuevo M. S., Cortizo M. S. & Cortizo A. M. Development of an osteoconductive PCL-PDIPF-hydroxyapatite composite scaffold for bone tissue engineering. J. Tissue. Eng. Regen. M. 5, e126–e135 (2011). 21312338 10.1002/term.394

[b15] Nikpour M. R., Rabiee S. M. & Jahanshahi M. Synthesis and characterization of hydroxyapatite/chitosan nanocomposite materials for medical engineering applications. Compos. Part. B-Eng. 43, 1881–1886 (2012).

[b16] Coldea A., Swain M. V. & Thiel N. Mechanical properties of polymer-infiltrated-ceramic-network materials. Dent. Mater. 29, 419–426 (2013). 23410552 10.1016/j.dental.2013.01.002

[b17] Fereshteh Z., Mallakpour F., Fathi M., Mallakpour S. & Bagri A. Surface modification of Mg-doped fluoridated hydroxyapatite nanoparticles using bioactive amino acids as the coupling agent for biomedical applications. Ceram. Int. 41, 10079–10086 (2015).

[b18] Tham W. L., Chow W. S. & Ishak Z. A. M. Effects of titanate coupling agent on the mechanical, thermal, and morphological properties of poly (methyl methacrylate)/hydroxyapatite denture base composites. J. Compos. Mater. 45, 2335–2345 (2011).

[b19] Chow W. S., Tham W. L. & Ishak Z. A. M. Improvement of Microstructure and Properties of Poly (methyl methacrylate)/Hydroxyapatite Composites Treated with Zirconate Coupling Agent. J. Thermoplast. Compos. 25, 165–180 (2012).

[b20] Wu T. . Adsorption characteristics of acrylonitrile, p-toluenesulfonic acid, 1-naphthalenesulfonic acid and methyl blue on graphene in aqueous solutions. Chem. Eng. J. 173, 144–149 (2011).

[b21] Zhou X. . Ultra-small graphene oxide functionalized with polyethylenimine (PEI) for very efficient gene delivery in cell and zebrafish embryos. Nano Res. 5, 703–709 (2012).

[b22] Rastogi R. . Comparative study of carbon nanotube dispersion using surfactants. J. Colloid Interf. Sci. 328, 421–428 (2008). 10.1016/j.jcis.2008.09.015 18848704

[b23] Moon I. K. . 2D graphene oxide nanosheets as an adhesive over-coating layer for flexible transparent conductive electrodes. Sci. Rep-UK 3, 1112 (2013).

[b24] Lai Y. . Graphene oxide as nanocarrier for sensitive electrochemical immunoassay of clenbuterol based on labeling amplification strategy. Talanta 107, 176–182 (2013). 23598209 10.1016/j.talanta.2013.01.002

[b25] Wang Y. . Electrocatalytic oxidation and detection of N-acetylcysteine based on magnetite/reduced graphene oxide composite-modified glassy carbon electrode. Electrochim. Acta 111, 31–40 (2013).

[b26] Goto T., Kim I. Y., Kikuta K. & Ohtsuki C. Hydroxyapatite formation by solvothermal treatment of α-tricalcium phosphate with water-ethanol solution. Ceram. Int. 38, 1003–1010 (2012).

[b27] Rulis P., Yao H., Ouyang L. & Ching W. Y. Electronic structure, bonding, charge distribution, and x-ray absorption spectra of the (001) surfaces of fluorapatite and hydroxyapatite from first principles. Phy. Rev. B 76, 245410 (2007).

[b28] Shuai C., Gao C., Feng P. & Peng S. Graphene-reinforced mechanical properties of calcium silicate scaffolds by laser sintering. RSC Adv. 4, 12782–12788 (2014).

[b29] Zhang W. L., Liu Y. D. & Choi H. J. Graphene oxide coated core–shell structured polystyrene microspheres and their electrorheological characteristics under applied electric field. J. Mater. Chem. 21, 6916–6921 (2011).

[b30] Li Z. F. . Covalently-grafted polyaniline on graphene oxide sheets for high performance electrochemical supercapacitors. Carbon 71, 257–267 (2014).

[b31] Xu Y., Wu Q., Sun Y., Bai H. & Shi G. Three-dimensional self-assembly of graphene oxide and DNA into multifunctional hydrogels. ACS Nano 4, 7358–7362 (2010). 21080682 10.1021/nn1027104

[b32] Yang Y. . Construction of a graphene oxide based noncovalent multiple nanosupramolecular assembly as a scaffold for drug delivery. Chem-Eur. J. 18, 4208–4215 (2012). 22374621 10.1002/chem.201103445

[b33] Cui H. . Facile synthesis of graphene oxide-enwrapped Ag_3_PO_4_ composites with highly efficient visible light photocatalytic performance. Mater. Lett. 93, 28–31 (2013).

[b34] Hwa K. Y. & Subramani B. Synthesis of zinc oxide nanoparticles on grapheme-carbon nanotube hybrid for glucose biosensor applications. Biosens. Bioelectron. 62, 127–133 (2014). 24997365 10.1016/j.bios.2014.06.023

[b35] Li G., Shi L., Zeng G., Zhang Y. & Sun Y. Efficient dehydration of the organic solvents through graphene oxide (GO)/ceramic composite membranes. RSC Adv. 4, 52012–52015 (2014).

[b36] Wu J. . Enhanced mechanical and gas barrier properties of rubber nanocomposites with surface functionalized graphene oxide at low content. Polymer 54, 1930–1937 (2013).

[b37] Suk J. W., Piner R. D., An J. & Ruoff R. S. Mechanical properties of monolayer graphene oxide. ACS Nano 4, 6557–6564 (2010). 20942443 10.1021/nn101781v

[b38] Wang J. . O-(Carboxymethyl)-chitosan nanofiltration membrane surface functionalized with graphene oxide nanosheets for enhanced desalting properties. ACS Appl. Mater. Inter. 7, 4381–4389 (2015). 10.1021/am508903g 25635511

[b39] Ren H. . Competitive adsorption of dopamine and rhodamine 6G on the surface of graphene oxide. ACS Appl. Mater. Inter. 6, 2459–2470 (2014). 10.1021/am404881p 24494630

[b40] Shuai C. . Graphene oxide reinforced poly (vinyl alcohol): nanocomposite scaffolds for tissue engineering applications. RSC Adv. 5, 25416–25423 (2015).

[b41] Bitounis D., Ali-Boucetta H., Hong B. H., Min D. H. & Kostarelos K. Prospects and challenges of graphene in biomedical applications. Adv. Mater. 25, 2258–2268 (2013). 23494834 10.1002/adma.201203700

[b42] Ramesh S., Ramesh K. & Arof A. K. Fumed silica-doped poly (vinyl chloride)-poly (ethylene oxide)(PVC/PEO)-based polymer electrolyte for lithium ion battery. Int. J. Electrochem. Sc. 8, 8348–8355 (2013).

[b43] Song J. M., Shin J., Sohn J. Y. & Nho Y. C. Ionic aggregation characterization of sulfonated PEEK ionomers using by X-ray and DMA techniques. Macromol. Res. 20, 477–483 (2012).

[b44] Zhang G., Yu H., Zhang C., Liao H. & Coddet C. Temperature dependence of the tribological mechanisms of amorphous PEEK (polyetheretherketone) under dry sliding conditions. Acta Mater. 56, 2182–2190 (2008).

[b45] Ma R. . Preparation, characterization, *in vitro* bioactivity, and cellular responses to a polyetheretherketone bioactive composite containing nanocalcium silicate for bone repair. ACS Appl. Mater. Inter. 6, 12214–12225 (2014). 10.1021/am504409q 25013988

[b46] He J., Yang X., Mao J., Xu F. & Cai Q. Hydroxyapatite-poly (l-lactide) nanohybrids via surface-initiated ATRP for improving bone-like apatite-formation abilities. Appl. Surf. Sci. 258, 6823–6830 (2012).

[b47] Zheng Y., Xiong C. & Zhang L. Formation of bone-like apatite on plasma-carboxylated poly (etheretherketone) surface. Mater. Lett. 126, 147–150 (2014).

[b48] Adams L. A. & Essien E. R. *In Vitro* Transformation of Sol-gel Derived Bioactive Glass from Sand. Am. J. Biomed. Sci. 7, 218–228 (2015).

[b49] Stares S. L., Fredel M. C., Greil P. & Travitzky N. Paper-derived hydroxyapatite. Ceram. Int. 39, 7179–7183 (2013).

[b50] Li R., Liu C. & Ma J. Studies on the properties of graphene oxide-reinforced starch biocomposites. Carbohyd. Polym. 84, 631–637 (2011).

[b51] Xu Y., Hong W., Bai H., Li C. & Shi G. Strong and ductile poly (vinyl alcohol)/graphene oxide composite films with a layered structure. Carbon 47, 3538–3543 (2009).

[b52] Wu W. Z. . Manufacture and thermal deformation analysis of semicrystalline polymer polyether ether ketone by 3D printing. Mater. Res. Innov. 18, 12–16 (2014).

[b53] Garcia-Gonzalez D., Rusinek A., Jankowiak T. & Arias A. Mechanical impact behavior of polyether-ether-ketone (PEEK). Compos. Struct. 124, 88–99 (2015).

[b54] Rinawa K., Maiti S. N., Sonnier R. & Cuesta J. M. L. Influence of microstructure and flexibility of maleated styrene-b-(ethylene-co-butylene)-b-styrene rubber on the mechanical properties of polyamide 12. Polym. Bull. 71, 1131–1152 (2014).

[b55] Depan D., Girase B., Shah J. S. & Misra R. D. K. Structure-process-property relationship of the polar graphene oxide-mediated cellular response and stimulated growth of osteoblasts on hybrid chitosan network structure nanocomposite scaffolds. Acta Biomater. 7, 3432–3445 (2011). 21664303 10.1016/j.actbio.2011.05.019

[b56] Luo Y. . Enhanced proliferation and osteogenic differentiation of mesenchymal stem cells on graphene oxide-incorporated electrospun poly (lactic-co-glycolic acid) nanofibrous mats. ACS Appl. Mater. Inter. 7, 6331–6339 (2015). 10.1021/acsami.5b00862 25741576

[b57] Jing X. . Preparation of thermoplastic polyurethane/graphene oxide composite scaffolds by thermally induced phase separation. Polym. Composite. 35, 1408–1417 (2014).

[b58] Kim J. . Bioactive effects of graphene oxide cell culture substratum on structure and function of human adipose-derived stem cells. J. Biomed. Mater. Res. A 101, 3520–3530 (2013). 23613168 10.1002/jbm.a.34659

[b59] Liao K. H., Lin Y. S., Macosko C. W. & Haynes C. L. Cytotoxicity of graphene oxide and graphene in human erythrocytes and skin fibroblasts. ACS Appl. Mater. Inter. 3, 2607–2615 (2011). 10.1021/am200428v 21650218

[b60] Kumar S. & Chatterjee K. Strontium eluting graphene hybrid nanoparticles augment osteogenesis in a 3D tissue scaffold. Nanoscale 7, 2023–2033 (2015). 25553731 10.1039/c4nr05060f

[b61] Wang D. X. . Enhancing the bioactivity of Poly (lactic-co-glycolic acid) scaffold with a nano-hydroxyapatite coating for the treatment of segmental bone defect in a rabbit model. Int. J. Nanomed. 8, 1855–1865 (2013). 10.2147/IJN.S43706 PMC3656818 23690683

[b62] Dinarvand P. . New approach to bone tissue engineering: simultaneous application of hydroxyapatite and bioactive glass coated on a poly (L-lactic acid) scaffold. ACS Appl. Mater. Inter. 3, 4518–4524 (2011). 10.1021/am201212u 21999213

[b63] Chen J., Li Y., Huang L., Jia N., Li C. & Shi G. Size fractionation of graphene oxide sheets via filtration through track-etched membranes. Adv. Mater. 27, 3654–3660 (2015). 25960040 10.1002/adma.201501271

[b64] Zhang M., Wang Y., Huang L., Xu Z., Li C. & Shi G. Multifunctional pristine chemically modified graphene films as strong as stainless steel. Adv. Mater. 27, 6708–6713 (2015). 26422818 10.1002/adma.201503045

[b65] Chen J. . Synthesis of graphene oxide sheets with controlled sizes from sieved graphite flakes. Carbon 110, 34–40 (2016).

[b66] Shuai C. . Nano-hydroxyapatite improves the properties of β-tricalcium phosphate bone scaffolds. Int. J. Appl. Ceram. Tec. 10, 1003–1013 (2013).

[b67] Kokubo T., Kushitani H., Sakka S., Kitsugi T. & Yamamuro T. Solutions able to reproduce *in vivo* surface-structure changes in bioactive glass-ceramic A-W. J. Biomed. Mater. Res. 24, 721–734 (1990). 2361964 10.1002/jbm.820240607

