# Supplementary material for: Graphene oxide as an interface phase between polyetheretherketone and hydroxyapatite for tissue engineering scaffolds
Source: Sci Rep. 2017 Apr 20;7:46604. doi: 10.1038/srep46604 (PMC5397874; doi:10.1038/srep46604)
Supplement: Supporting Information [file srep46604-s1.pdf]

Graphene oxide as an interface phase between polyetheretherketone and hydroxyapatite for tissue engineering scaffolds

Shuping Peng[1,2,3,#], Pei Feng[4,#], Ping Wu[6], Wei Huang[4], Youwen Yang[4], Wang Guo[4], Chengde Gao[4], Cijun Shuai[4,5,*]

Supporting information

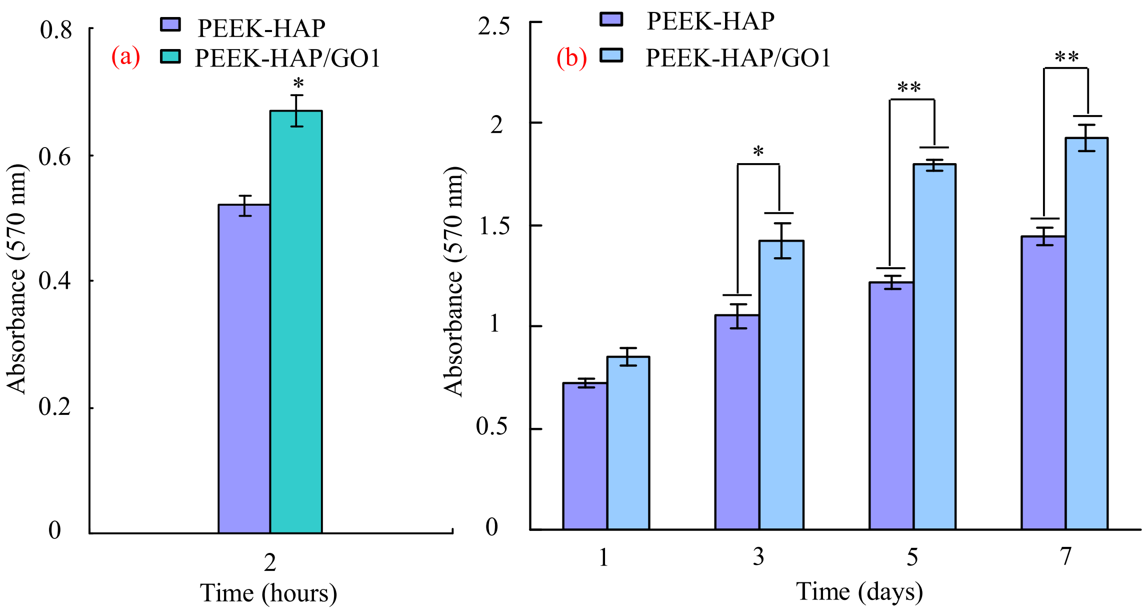

Figure S1. MG-63 cells adhesion and proliferation. Adhesion of MG-63 cells on the scaffolds at 2 hours after culture (a), proliferation of MG-63 cells on the scaffolds for 1 day, 3 days, 5 days and 7 days. The data represent the mean ± SD (n=6). *, statistically significant difference ($P < 0.05$); **, very significant difference ($P < 0.01$).

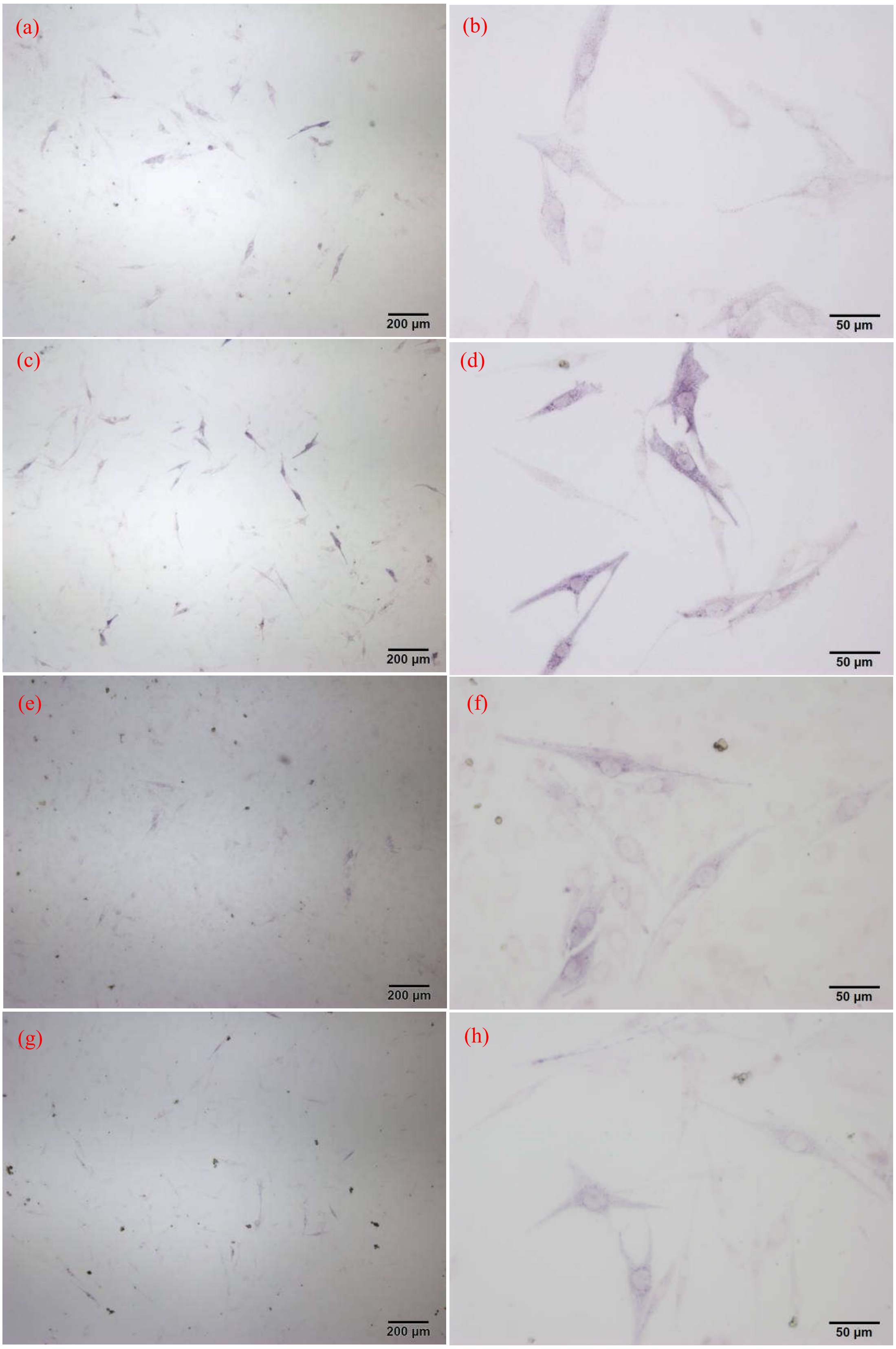

Figure S2. ALP staining images. MG-63 cells after culturing on the PEEK-HAP/GO1 scaffolds for 1 day (a and b), 3 days (c and d), 5 days (e and f) and 7 days (g and h).

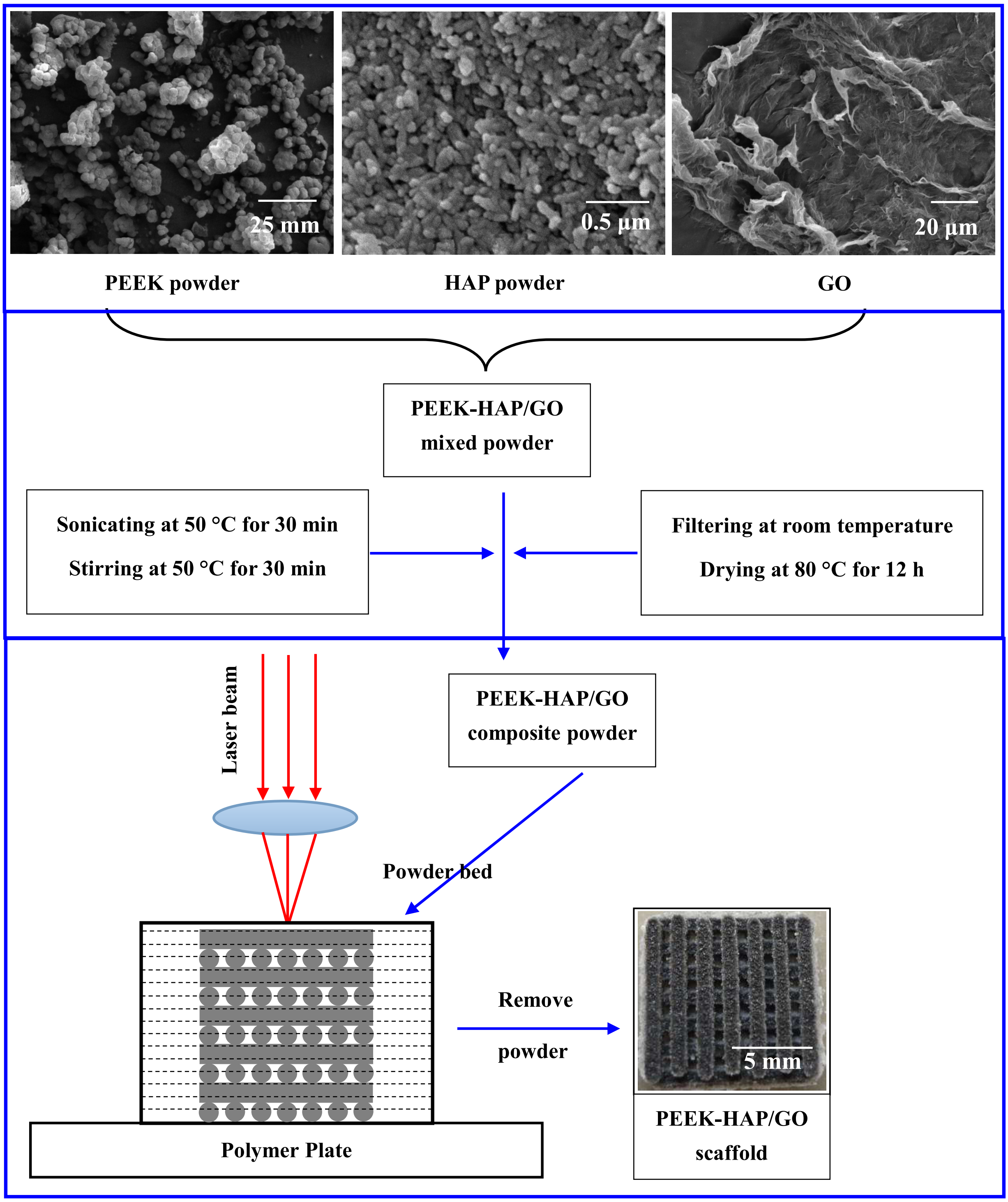

Figure S3. Schematic of the procedure used to fabricate PEEK-HAP/GO scaffold.
